# Transcranial Magnetic Stimulation of the Right Superior Parietal Lobule Modulates the Retro-Cue Benefit in Visual Short-Term Memory

**DOI:** 10.3390/brainsci11020252

**Published:** 2021-02-18

**Authors:** Fabiano Botta, Juan Lupiáñez, Valerio Santangelo, Elisa Martín-Arévalo

**Affiliations:** 1 Department of Experimental Psychology and Mind, Brain, and Behavior Research Center (CIMCYC), University of Granada, 18011 Granada, Spain; jlupiane@ugr.es (J.L.); emartina@ugr.es (E.M.-A.); 2 Department of Philosophy, Social Sciences & Education, University of Perugia, 06123 Perugia, Italy; valerio.santangelo@unipg.it; 3 Neuroimaging Laboratory, IRCCS Santa Lucia Foundation, 00179 Rome, Italy

**Keywords:** visual short-term memory, superior parietal lobe (SPL), superior longitudinal fasciculus (SLF), transcranial magnetic stimulation (TMS), spatial attention

## Abstract

Several studies have shown enhanced performance in change detection tasks when spatial cues indicating the probe’s location are presented after the memory array has disappeared (i.e., retro-cues) compared with spatial cues that are presented simultaneously with the test array (i.e., post-cues). This retro-cue benefit led some authors to propose the existence of two different stores of visual short-term memory: a weak but high-capacity store (fragile memory (FM)) linked to the effect of retro-cues and a robust but low-capacity store (working memory (WM)) linked to the effect of post-cues. The former is thought to be an attention-free system, whereas the latter would strictly depend on selective attention. Nonetheless, this dissociation is under debate, and several authors do not consider retro-cues as a proxy to measure the existence of an independent memory system (e.g., FM). We approached this controversial issue by altering the attention-related functions in the right superior parietal lobule (SPL) by transcranial magnetic stimulation (TMS), whose effects were mediated by the integrity of the right superior longitudinal fasciculus (SLF). Specifically, we asked whether TMS on the SPL affected the performance of retro cues vs. post-cues to a similar extent. The results showed that TMS on the SPL, mediated by right SLF-III integrity, produced a modulation of the retro-cue benefit, namely a memory capacity decrease in the post-cues but not in the retro-cues. These findings have strong implications for the debate on the existence of independent stages of visual short-term memory and for the growing literature showing a key role of the SLF for explaining the variability of TMS effects across participants.

## 1. Introduction

Classical theories of visual short-term memory (VSTM) usually distinguish between two main systems: working memory, which is a low-capacity but durable memory system that lasts seconds to minutes and is robust enough to prevent visual interference, [1] and visual sensory memory, better known as iconic memory, which is a highly detailed but brief memory system that lasts around 500 ms and is not resistant to visual masking [2,3].

In typical visual working memory (WM) paradigms, such as change detection tasks [1], participants are presented with a memory array of visual items. After a retention interval, a test array is presented, and participants are asked to determine whether one of the items has changed or not. Working memory capacity in these conditions typically does not surpass the limit of 3–4 items. However, previous studies have shown that task execution significantly improves when a cue indicates which one of the memory items will be probed in the test display during the retention interval [4,5]. Given that this type of cue is presented after the memory array has disappeared, its effect is retrospective, and for this reason, it is usually called a retro-cue.

One of the interpretations of the so-called retro-cue benefit is that it results from a fragile but large-capacity visual short-term memory store (i.e., fragile memory (FM)), which would be an intermediate system between the iconic and visual working memory (WM) [5,6,7]. More specifically, the multiple store theory [6,7] hypothesizes that the lower task performance in change detection paradigms in the absence of retro-cue trials (i.e., when the memory probe is presented simultaneously or right after the test array in the so-called post-cue trials) [7] reflects the visual WM. This can be defined as a durable and robust but low-capacity storage system whose content would represent the information that has resisted the visual masking produced by the test display via endogenous attentional mechanisms [8]. On the other hand, performance in the retro-cue trials would gauge a high-capacity short-term memory system that stores the existing visual representations before the masking produced by the test array, and it is therefore fragile against visual interference. FM, which is considered a sensory memory store [9], would have a lower—but still high—capacity than iconic memory but a longer duration (up to 4 s), and it would be resistant to light masks [6].

The multiple store theory postulates a differential impact of the attention selection mechanisms in the two memory stores. More specifically, this theory proposes that FM traces are formed without the need for selective attention, while visual WM traces actually depend on selective attention to be created. Retro-cues would be just a tool to report, but not to build, the fragile memory representations. This assumption has been defended by Vandenbroucke et al. [7], who manipulated the availability of attentional resources during the encoding phase of a change detection task by either increasing the temporal uncertainty about the moment in which the memory array would be presented or by requiring participants to simultaneously perform another task. They showed that attentional diversion at encoding produced more costs in the post-cue trials, which were supposed to measure WM, than in the retro-cue trials, which were supposed to assess FM, wherein performance was slightly but reliably affected. These results were interpreted as an index of a structural dissociation between WM, whose content would be actively maintained by selective spatial attention through the recurrent activation of the visual and frontoparietal regions [10,11,12], and FM, wherein visual icons would be maintained in visual cortical area V4 [13] independent of the attentional focus.

Even though the multiple store theory conceptualizes retro- and post-cue trials as two different tools to gauge two independent memory systems—the formation of which is considered independent of and dependent on attentional processes, respectively—this dissociation is not unanimously accepted. Many studies (e.g., see Souza and Oberauer [14] for a review) have, in fact, extensively considered subtraction between retro and post-cue trials (i.e., the retro-cue benefit) as a way to investigate how focusing attention retrospectively affects WM representations, thus not considering retro-cues as a proxy to prove the existence of FM as an independent memory system free of attention [15]. Souza and Oberauer [14] suggested that retro-cues simply enhance the allocation of attention to VSTM representations, thus strengthening and stabilizing individual items against perceptual interference. Moreover, despite the multiple store theory supporting the hypothesis that retro-cues protect memory representations from perceptual interference, according to Souza and Oberauer [14], it is possible to assume a single WM system wherein representations can be stored with a different degree of robustness, and focusing attention on them increases their robustness.

While the existence of FM as an independent and attention-free memory system is under debate [16,17,18,19], the intimate interplay between selective attention and visual WM has been largely demonstrated by a huge number of studies [20,21]. As suggested by several authors [22,23,24], the neural substratum supporting this interaction might correspond to the dorsal frontoparietal attention network [25], whose main role would be to trigger and sustain endogenous control of attention signals over time in order to bias the processing of task-relevant stimulus features and locations along the sensory cortex on the basis of the current task’s goals [26].

Crucially, involvement of the dorsal frontoparietal attention network has been consistently observed during WM tasks [12,23], thus strengthening the idea that attention and WM share a common substrate [22,23,24,27,28,29]. The dorsal frontoparietal attentional network bilaterally involves the superior parietal lobule (SPL), the intraparietal sulcus (IPS) and the frontal eye fields (FEFs) [26,30]. Moreover, white matter connectivity studies [31,32,33] highlighted the role of the superior longitudinal fasciculus (SLF) as the most important white matter tract connecting the above-mentioned parietal and frontal attentional areas [31]. Importantly, the integrity of the SLF—particularly the first and the third branches (SLF-I and SLF-III)—has been reliably associated with the efficiency of attentional orienting, both in healthy participants and brain-damaged patients [34,35,36]. Moreover, previous transcranial magnetic stimulation (TMS) experiments have also shown that the integrity of the SLF represents an important factor to attenuate the temporal modulation produced by the TMS on the activity of the attentional regions [37,38,39,40], with larger TMS effects on participants with lower integrities of the SLF.

### Aims of the Present Study

In the present study, we aimed to investigate through a causal approach the role of selective spatial attention in visual short-term memory with vs. without retrospective prioritization. More specifically, we asked whether and how endogenous top-down spatial attention might dissociate the performance in retro-cue trials (hypothetically gauging FM) from the performance in post-cue trials (hypothetically gauging WM). We applied online TMS to the right SPL to interfere with attentional orienting while measuring participants’ memory capacities in a change detection task with retro-cue vs. post-cue trials. TMS pulses were applied online, either to the right SPL or to an active control site (the vertex). The right SPL was selected, as this area has consistently been involved in the endogenous control of attentional orienting toward both external (perceptual) and internal (WM) objects [11]. Previous TMS studies have also shown that repetitive TMS (rTMS) over the right posterior parietal cortex (PPC), including the SPL, interferes with visuo-spatial WM, whereas rTMS over the left PPC does not [41,42,43].

**Figure 1:**
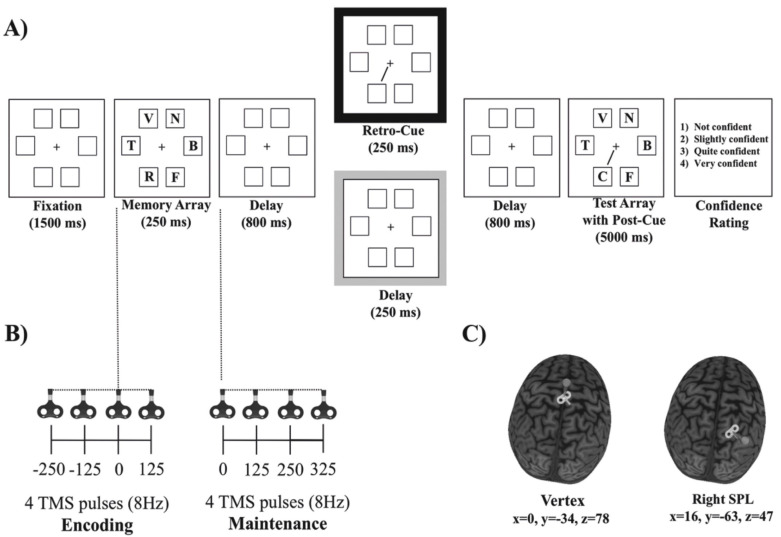
(**A**) Sequence of the events in a given trial. The black and gray boxes represent retro-cue and post-cue trials, respectively. (**B**) Transcranial magnetic stimulation (TMS) protocol: a burst of four TMS pulses at 8 Hz, administered either during the encoding phase or during the maintenance phase. (**C**) TMS sites: vertex (left panel) and right superior parietal lobe (SPL) (right panel).

To specifically understand how and whether the temporal modulation of the attentional function produced by SPL stimulation affects the encoding or maintenance phases, TMS pulses were delivered either before or after the memory array presentation (see magnetic resonance imaging (MRI) and TMS protocol and Figure 1B). Since previous studies have shown strong influence by the SLF on the modulation of visual attention and WM [44,45,46,47], as well as over the impact of TMS on affecting performance in attentional tasks [37,38,39,40], the integrity of the right SLF was included in the data analysis as a likely modulator of the observed results.

We hypothesized that if FM traces were formed and maintained independently of focal attention, then the disruption of the activity of the right SPL should only affect the memory content in the post-cue trials, when endogenous attention—at encoding and maintenance—is supposed to be necessary to protect the memory traces from interference, leaving the performance unaffected in the retro-cue trials. Furthermore, TMS modulation of the right SPL attentional function is specifically expected from participants with reduced integrity of the right SLF (either in right SLF-III, SLF-I or in both), who should be more prone to TMS effects. Data will be analyzed and discussed, taking into account both the multiple store theory approach [7] by comparing retro vs. post-cues and the theory of the single WM store [14] by analyzing the modulation of the retro-cue benefit (the difference between retro-cue and post-cue trials).

## 2. Materials and Methods

### 2.1. Participants

A total of 17 right-handed, healthy volunteers (8 women, with a mean age of 25.5 years, SD = 4.3 years) took part in the study. The sample size was established on the basis of the previous literature, employing a similar sample size and obtaining reliable effects [6,7,19,48] and power analysis, performed with the G*Power 3.1.9.2 program [48] on the data from a behavioral pilot study conducted with 16 participants, which showed that with an alpha level = 0.05 and a beta level = 0.8, a minimum of 11 participants was needed to obtain a large effect size. All participants did not know the aim of the study, which lasted for approximately 60 min. They had normal or corrected-to-normal visual acuity, normal color discrimination and no history of head injuries or neurological or psychiatric problems. All participants were tested before the experimental session for TMS and magnetic resonance imaging (MRI) exclusion criteria [49]. Participants signed an informed consent form to participate for monetary compensation (10 euros/hour). They were informed that they were free to abandon the experiment whenever they wanted without being penalized. Data from two participants were excluded from the analyses due to technical problems in MRI data acquisition, leading to a final sample of 15 participants (6 women, mean age of 25.5 years, SD = 4.5 years). The experiment was conducted following the ethical guidelines of the University of Granada and in accordance with the ethical standards of the 1964 Declaration of Helsinki (last update: Seoul, 2008) as part of a research project approved by the University of Granada Ethical Committee (536/CEIH/2018).

### 2.2. Apparatus and Stimuli

The experiment was run on a computer with a 1 GHz Pentium III processor. The stimuli were presented and the data were acquired by E-prime software [50]. The stimuli appeared on a light gray background on a 19-inch color VGA monitor (Benq T903, 19″ wide, 1280 × 1024, 60 Hz). The distance between the video monitor and the head of the participant was approximately 80 cm. A 0.61° × 0.61° fixation cross was continuously displayed at the center of the screen. The memory and test arrays consisted of six 0.93° × 0.93° letters presented inside 1.2° × 1.2° placeholders (see Figure 1). The placeholders consisted of squares evenly spaced around an imaginary circle, centered at fixation with a radius of approximately 3°. The letters were uppercase and were randomly picked from the BCDFGHJKLNPQRSTVZ set (i.e., all the consonants of the Spanish alphabet except M, Ñ and W, which were not used in order to avoid possible confusion between similar letters). The retro-cue and the post-cue were black lines (0.06° × 1.6°) that pointed from the central fixation to one of the six possible placeholders.

### 2.3. Behavioral Procedure

The behavioral procedure (see Figure 1) was similar to the one used in Botta et al. [16]. Participants were seated on a comfort TMS robot’s armchair (http://www.axilumrobotics.com/en/, accessed on 30 June 2019) and asked to maintain their gaze on the central cross during all of the trial. At the beginning of each trial, six placeholders and a central red fixation cross that turned black after 500 ms were presented. After 1000 ms, the six letters constituting the memory array were then presented for 250 ms within the placeholders. The participants were required to remember as many letters as possible. We tested the participants’ memory for letters instead of line orientations to minimize the grouping or chunking effects [15], increase the task’s difficulty [6] and consequently decrease the likelihood of ceiling effects. After the offset of the memory display, a blank retention display was presented for 800 ms. Up to this time point, there were no differences between conditions. Then, for the retro-cue trials, a 250 ms and 100% predictive spatial cue indicating the location of the potentially changing item was presented. After another blank display with 800 ms of delay, the test array, including the spatial (post) cue, was presented for 5000 ms or until a response was given. For the post-cue trials, no retro-cue was presented, but rather a 250 ms blank display. Then, for these trials, only the second spatial cue (i.e., the post-cue) was presented, along with the test array. Therefore, the retro-cue and post-cue trials only differed in the presentation of the retro-cue 800 ms after the memory array offset. The participants had to press with their left hand one of two keys located on the left side of the keyboard in order to report whether or not the letter indicated by the retro-cue or by the post-cue matched the letter presented at the corresponding location in the memory array. The probed letter changed in 50% of the trials. After each response, participants were required to use the mouse with their right hand to establish the degree of confidence about their objective response: (1) not confident, (2) slightly confident, (3) quite confident, and (4) very confident.

Since previous research suggested that training participants to use a retro-cue was necessary to guarantee a proper measure of fragile memory [19], participants received a training session before the experiment. Specifically, prior to the start of the training session, participants performed 2 blocks of 12 practice trials. In the first block, the memory array was presented for 1000 ms, and participants received visual feedback on their response accuracy. If necessary, the first practice block was repeated until participants fully understood how to perform the task. In the second practice block, participants were presented with trials identical to the training trials. The training session consisted of 2 blocks of 60 trials. The experiment was then performed in a separate session right after training and consisted of 384 trials (48 trials for each combination of cue type (retro-cue vs. post-cue), stimulation site (right SPL vs. vertex) and stimulation time window (encoding vs. maintenance)).

### 2.4. MRI and TMS Protocol

Structural T1-weighted anatomical magnetic resonance (MR) scans of all participants were acquired at the Mind, Brain and Behavior Research Center (CIMCYC) at the University of Granada. We used a 3-T Siemens magnetization-prepared rapid gradient echo (flip angle = 7, repetition time (RT) = 2530 ms, echo time (TE) = 3.5 ms, slice thickness = 1 mm, and field of view (FOV) = 256 mm). Prior to the TMS experimental session, we determined the hotspot for the first dorsal interosseous (FDI), defined as the optimum site over the primary motor cortex (M1) which evoked the highest contralateral motor evoked potentials (MEPs) in the relaxed FDI. Afterward, we calculated the individual resting motor threshold (rMT), which was the minimum stimulus intensity eliciting MEPs >50 μV in five out of ten consecutive trials [51,52,53]. Electromyography (EMG) and MEPs were recorded from the left FDI using snap surface electrodes (Natus Neurology, Middleton, WI, United States). Focal TMS over the right M1 was performed with a 70 mm TMS figure-of-eight, connected to a biphasic stimulator (Super Rapid 2, Magstim 2002, Whitland, UK) and tangentially located on the scalp at an angle of approximately 45° from the midline [54].

In each trial, we applied a burst of four TMS pulses at 8 Hz either during the encoding phase (250 ms and 125 ms before the memory array, simultaneous with the onset of the memory array (0 ms), and 125 ms after the onset of the memory array) or during the maintenance phase at 0, 125, 250 and 375 ms after the onset of the first blank retention interval (see Figure 1). This protocol was similar to others previously used with similar stimulation parameters [55,56], allowing us to cover a long temporal range either during (and linked to) encoding or maintenance and keeping well within the published safety limits for TMS [49]. During the experiment, we stimulated each participant at 120% of her or his rMT (mean intensity = 67% of the maximum stimulator output (MSO) in the total sample (SD = 8.3)).

The scalp coordinates of the stimulation sites were localized by using the native space of each participant’s T1-weighted anatomical MRI scans. The TMS coil was controlled by a robotic arm (TMS Robot, Axilum Robotics, http://www.axilumrobotics.com/en/, accessed on 30 June 2019) and a TMS neuronavigation system (Brainsight, Rogue Systems, Montreal, QC, Canada), which allowed us to estimate and track in real time the relative position, orientation and tilting of the coil on the sectional and 3D reconstructions of the participants’ MRIs with a precision of 5 mm. The TMS robot warranted the precise stimulation of the selected brain regions (the right SPL and the vertex) by automatically readjusting the position of the coil if a movement larger than 5 mm was detected.

The selected TMS stimulation site (see Figure 1) corresponded to MNI coordinates of *x* = 16, *y* = −63 and *z* = 47 (right superior parietal gyrus and lobe and right SPL [57]). The control stimulation site was the vertex (MNI coordinates of *x* = 0, *y* = −34 and *z* = 78 [58]), which was not expected to induce any specific behavioral effects based on previous reports [59,60,61,62]. The right SPL and the vertex were stimulated in two sequential blocks, the order being counterbalanced across participants.

### 2.5. Diffusion Tensor Imaging (DTI) Analysis

DTI analyses were performed following the previously reported procedure (see Supplementary Methods in Thiebaut de Schotten et al. [31]). A total of 70 near-axial slices were acquired using a sequence of white matter fully optimized for DTI (based on spherical deconvolution [63]), providing isotropic (2 × 2 × 2 mm^3^) resolution and coverage of the whole head with a posterior–anterior phase of acquisition (echo time = 88 ms and repetition time = 8400 ms). At each slice location, 6 images were acquired with no diffusion gradient applied and 60 diffusion-weighted images, in which gradient directions were uniformly distributed in space. The diffusion weighting was equal to a b-value of 1500 s/mm^2^. In each slice, diffusion-weighted data were simultaneously registered and corrected for motion and geometrical distortion, adjusting the gradient accordingly (ExploreDTI: http://www.exploredti.com, accessed on 30 June 2019). Then, individual dissections of the tracts were carried out with TrackVis software (http://www.trackvis.org, accessed on 30 June 2019). Both branches (SLF-I and SLF-III) were isolated using a multiple region of interest (ROI) approach. Parietal and frontal ROIs (superior frontal region) and parietal and frontal ROIs (inferior frontal gyri) were delineated around the white matter for both the SLF-I and the SLF-III, respectively. A no-part ROI in the temporal white matter was also used to exclude streamlines of the arcuate fasciculus projecting to the temporal lobe [31,64]. Finally, the index employed as a surrogate for tract microstructural organization (i.e., mean hindrance modulated orientational anisotropy (HMOA) [63]) was extracted from each dissected tract (SLF-I and SLF-III) for both the right and left hemisphere. It should be noted that the left SLF-I and SLF-III were used as controls for the right SLF’s specificity. The mean HMOA is defined as the absolute amplitude of each lobe of the fiber orientation distribution and is considered highly sensitive to the axonal myelination, fiber diameter and axonal density [63].

### 2.6. Design and Data Analysis

The behavioral performance was analyzed by calculating the memory capacity K index of each participant by using Cowan’s K formula [65]: K = [(% hits on change trials − chance) + (% correct rejections on no-change trials − chance) × current set size. A powerful aspect of the K index is related to the intrinsic correction for guessing, different from the percentages of response accuracy [8]. Data points two standard deviations (SDs) below or above the mean value of each possible combination of cue type, stimulation site and stimulation time window were considered outliers (5% of trials overall) and replaced by values thresholded at ± 2 SDs to the mean value of that combination [66]). This procedure allowed us to avoid our results being biased by extreme (i.e., outlier) values [67].

All statistical analyses were conducted by using linear mixed effect models (LMMs), which constitute a statistically powerful method that takes into account both fixed factors (the variables of interest) and random effects (variation that is not explained by the variables of interest). In particular, one of the advantages of the linear mixed model is that it resolves the issue of independence among repeated measures by controlling for individual variation among the participants [68]. In other words, LMMs not only estimate the main effects and interactions between fixed effect parameters (i.e., the ones that are manipulated, as is done by traditional ANOVAs), but also estimate and simultaneously control for the variance and covariance components of random effects caused by inter-participant variability.

Thus, we constructed linear mixed models using the lmer function of the lme4 R package, version 1.1–21 [69], with participants as the random factor and the cue type (retro-cue vs. post-cue), stimulation site (right SPL vs. vertex) and stimulation time window (encoding vs. maintenance) as the fixed factors. To assess the contribution of the integrity of the right SLF, the HMOA index of both the right and left SLF-III and SLF-I were also included as fixed factors in two different models for both memory capacity and confidence ratings, considering the left HMOA indexes as a control for the expected right SLF-specific modulations. More specifically, the fixed full structure of each model was composed of two four-way interactions (cue-type × stimulation side × stimulation time window × right SLF + cue-type × stimulation side × stimulation time window × left SLF).

To determine the best structure for the random and fixed components, we followed a well-known procedure [70]. Specifically, as a first step, for keeping the full fixed structure, we searched for the best random structure using restricted maximum likelihood (REML). Second, for keeping the established random structure, we searched for the best fixed structure using backward stepwise model comparison from the four-way interaction model to the main effect model. We selected the model with lower Bayesian information criteria (BIC) and significant χ2 tests for the log likelihood, using the maximum likelihood. The *p* values of the significant fixed effects were provided by the ANOVA function of the lmerTest R package, version 3.1–0 [71], using the REML. To follow up on four-way interactions, we divided the data into subsets according to the levels of the stimulation time window, while for three-way interactions, data were divided into subsets depending on the stimulation site. To qualify two-way interactions, we ran pairwise Bonferroni-corrected comparisons with the test interactions function of the phia R package [72]. Because our predictions were mainly related to the cue type (retro-cue vs. post-cue) and stimulation site (right SPL vs. vertex), for the sake of clarity, here we report and discuss only significant interactions involving at least these two factors. For a complete overview of the results, see the online supplementary material (Tables S1–S4).

## 3. Results

### 3.1. Retro-Cue vs. Post-Cue Analysis

**Figure 2:**
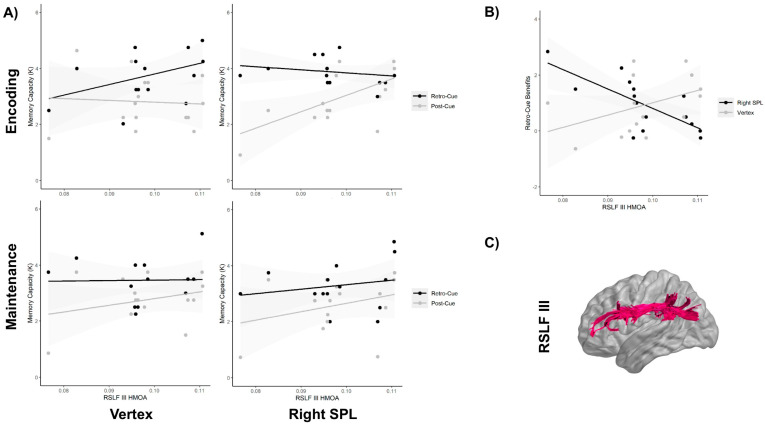
(**A**) Mean memory capacity (K) for each experimental condition of the cue type (retro-cue vs. post-cue; gray vs. black lines), stimulation site (right SPL vs. vertex; right vs. left panels) and stimulation time window (encoding vs. maintenance; upper vs. lower panels), depending on the hindrance modulated oriental anisotropy (HMOA) index of the right superior longitudinal fasciculus (RSLF-III). The shaded gray area around the lines is the 95% confidence interval. (**B**) Mean retro-cue benefits during the encoding phase, depending on the stimulation site (right SPL vs. vertex; black vs. gray lines). (**C**) Virtual in vivo dissection of the right SLF-III using deterministic tractography.

The analysis of the memory capacity showed a final model with significant four-way interaction between the cue type, stimulation site, stimulation time window and right SLF-III integrity (i.e., HMOA index) (F_1,90_ = 7.10, *p* = 0.009, η^2^_p_ = 0.072). To follow up on this four-way interaction, we first split the data by the stimulation time window. As can be observed in Figure 2, the three-way interaction between the cue type, stimulation site and right SLF-III integrity was not significant when the stimulation was applied at the maintenance phase (F_1,38_ = 0.06, *p* = 0.80, η^2^_p_ = 0.002) (Figure 2A, lower panels). In contrast, a highly significant three-way interaction was observed when the TMS was applied at the encoding phase (F_1,38_ = 12.6, *p* = 0.001, η^2^_p_ = 0.23) (Figure 2A, upper panels). Once more, to examine the significant three-way interaction, we divided the analysis by stimulation site. The analysis performed revealed a significant interaction between the cue type and the right SLF-III integrity when the right SPL was stimulated (F_1,12_ = 10.3, *p* = 0.007, η^2^_p_ = 0.39) (Figure 2A, upper right panel), but not when the vertex was stimulated (F_1,12_ = 0.57, *p* = 0.43, η^2^_p_ = 0.032) (see Figure 2A, upper left panel). Further models indicated that these interactions were not significantly affected by the order in which the right SPL and the vertex were stimulated, all *p*_s_ > 0.1. Post-hoc analysis revealed that when the right SPL was stimulated, the memory capacity in the post-cue trials decreased as a function of the reduced right SLF-III integrity (*p* = 0.018), while performance in the retro-cue trials remained unaffected (*p* = 0.56). It should be noted that the analysis did not show any association with the right SLF-I. Figure 2C represents the virtual in vivo dissection of the right SLF III using deterministic tractography. It is important to note that the sensitivity analysis demonstrated that when considering α = 0.05 and 1 − β = 0.80, the critical F for the highest interaction model with a numerator df of 1 and a denominator df of 90 was F = 3.94, which is indeed smaller than the one observed here for the four-way interaction between the cue type, stimulation site, stimulation time window and right SLF-III integrity (F_1,90_ = 7.10).

### 3.2. Retro-Cue Benefit Analysis

The analysis of the retro-cue benefit, calculated as the difference between the retro-cue trials and the post-cue trials, showed a significant three-way interaction between the stimulation site, stimulation time window and right SLF-III integrity (F_1,39_ = 9.1, *p* = 0.004, η^2^_p_ = 0.11). To follow up on this three-way interaction, we split the data by the stimulation time window. The analysis showed that the interaction between the stimulation site and the right SLF-III integrity was not significant when the stimulation was applied at the maintenance phase (F_1,13_ = 0.06, *p* = 0.80, η^2^_p_ = 0.002). A significant interaction between the stimulation site and the right SLF-III integrity was instead found when TMS was applied during the encoding phase (F_1,26_ = 12.6, *p* = 0.001, η^2^_p_ = 0.32). Bonferroni-corrected comparisons showed that vertex stimulation during encoding did not produce any modulation of the retro-cue benefit, depending on the right SLF-III integrity (*p* = 0.1), while SPL stimulation significantly increased the retro-cue benefits of those participants with lower right SLF-III integrities (*p* = 0.004) (Figure 2B). This result is interesting because it seems to paradoxically indicate that TMS produced an increase in retro-cue benefits when the integrity of the right SLF-III decreased. However, on the basis of the analysis performed in the previous section, it is important to highlight that this effect was mostly due to the TMS-driven decrease of the capacity in the post-cue trials, rather than an increase of the capacity in the retro-cue condition.

## 4. Discussion

In the present study, we used a causal approach to investigate whether and how endogenous top-down spatial attention might dissociate performance in retro-cue trials (supposedly gauging FM) from performance in post-cue trials (supposedly gauging WM). With this aim, while participants performed a change detection task measuring both types of short-term memories, we disrupted their attention with online TMS, applied to the right SPL (in comparison with a control area) during both the encoding and maintenance phases. We found that applying TMS over the right SPL during the encoding phase caused a decrease of the memory capacity in the post-cue condition, especially for those participants with lower right SLF-III integrities, while memory capacity in the retro-cue trials was unaffected. These findings are in good agreement with the previous literature showing that TMS effects are dramatically modulated by interindividual variability in white matter connectivity [32,37,38,39,40,73]. The HMOA index, used here to measure the integrity, has been shown to be strongly sensitive to the axonal density, myelination and diameter of the fibers [63], which are critical factors for the conduction speed across white matter tracts. Moreover, previous findings have also found that interindividual variability in performance can be related to the white matter characteristics underlying attentional networks [74,75]. In light of the above and related evidence [37,38,39,40], we consider that memory capacity in post-cue trials is protected against TMS stimulation in those participants with higher right SLF-III integrities, while participants with lower right SLF-III integrities are more strongly affected by TMS stimulation [37,38,73]. Note that we did not find any association with the right SLF-I. Given that the coordinate used for stimulating SPL seemed to also be part of the posterior intraparietal sulcus (right posterior IPS and SLP [57]), we speculate that the stimulation of this specific coordinate could have been mainly affected by the integrity of the SLF-III rather than the SLF-I.

In general, the present findings fit well with the multiple store theory [6], which proposes that retro-cueing is a way to assess a fragile short-term memory system separated from WM. More specifically, according to this theoretical model, fragile memory would be a sensory memory system subserved by the recurrent activity of the visual regions, particularly V4 [13], and unlike WM, it would be mostly independent of attention [7,9,19]. Consistent with this view, TMS modulation over the right SPL—one of the core nodes of the dorsal frontoparietal dorsal attentional network [25]—only affected the memory capacity in the post-cue trials (supposedly gauging WM), leaving the memory capacity in the retro-cue trials unaltered. Moreover, this effect depended on the right SLF-III integrity—the tract connecting the parietal lobe with the ventral premotor, ventral prefrontal and dorsolateral prefrontal cortex [47]—in agreement with theories highlighting the importance of the frontoparietal connections for conscious access of information in WM via attentional modulations [10,76].

However, if the results are observed from a different perspective by looking at the individual differences between the retro-cue and post-cue trials (the retro-cue benefit), another explanation that does not necessarily imply the existence of fragile memory as a system independent from spatial attention and WM is possible. As was previously mentioned, TMS over the right SPL led to a decrease in memory capacity in the post-cue condition associated with the right SLF-III integrity, along with an increase of the retro-cue benefit. Thus, a possible alternative interpretation of this result is that the reduction of the memory capacity in the post-cue trials produced by TMS stimulation during encoding was compensated by the action of the retro-cues. Previous research has indeed suggested that retro-cues boost the accessibility of short-term memory representations to levels higher than those acquired after encoding [14] by at least four hypothetical mechanisms which are under debate and not mutually exclusive: (1) freeing short-term memory capacity by removing irrelevant information (all the uncued items [77,78,79,80]); (2) strengthening the memory trace of the retro-cued item by improving the binding with its context [11,81,82,83]; (3) allowing retrieval to start ahead of testing, thus increasing the time for the accumulation of evidence in short-term memory before a response decision in comparison with the post-cue condition [84]; and (4) by protecting the retro-cued item from visual interference [6,85,86]. From this point of view, the retro-cue would prompt a series of processes that increase the likelihood of information being transferred into WM, rather than as a tool gauging an independent visual short-term memory system.

It could be speculated that post-cue trial performance likely depends on the initial distribution of the attentional resources at the encoding phase. Since the participants did not know which item would be probed until the presentation of the test array in the post-cue trials, attention in this condition should not be focused on any specific location but distributed rather homogeneously across the different placeholders. The previous literature has suggested that the attentional weight associated with memory array items and generated by spatial selection criteria are computed in the PPC [28,85,86,87]. Therefore, one possible interpretation of the present data is that modulation over the right SPL led to a general reduction of the attentional weights pre-assigned to each item location during encoding, thus decreasing their accessibility to consciousness and, consequently, their reportability. Since TMS was administered before the retro-cue presentation, and since the retro-cue and post-cue trials were identical up to that point, the alteration of the attentional function exerted by the right SPL during encoding must have been similar in both conditions. For this reason, it is plausible that the reduction of attentional resources during encoding produced by the stimulation was compensated by the action of the retro-cue, which could have boosted up and resurrected the cued item. This interpretation is consistent with previous studies showing that retro-cues could bring to consciousness sub-threshold targets which would otherwise have gone unperceived [88]. Coherently, the beneficial effect of the retro-cue was particularly strong in those participants who, due to a lower right SLF integrity, were more susceptible to being affected by TMS stimulation, and they were particularly weak in those participants in which a higher right SLF-III integrity could provide stronger protection from the temporal alteration of the right SPL.

Finally, the present findings show no TMS effect when the SPL was stimulated at the maintenance phase. Apparently, this result is in contrast with the attentional rehearsal theory [20], which suggests that the active maintenance of visuospatial information is accomplished by means of focal shifts of spatial attention toward memorized locations. However, it is possible that more frontal areas, such as the FEFs, are involved in rehearsal mechanisms during the maintenance phase, with the SPL activity being more related to the generation of endogenous attentional signals during the encoding phase. Consistent with this, it was suggested in Postle et al. [89] that even though the SPL is involved with attention-based rehearsal circuits, the source of the rehearsal mechanism could likely be located in more frontal areas, such as the FEFs and the dorsolateral prefrontal cortex (DLPFC). This fits well with previous data showing that TMS stimulation over the DLPFC during the maintenance phase produced a significant reduction in memory capacity in the post-cue trials but not in the retro-cue trials [8]. Future studies should more directly test this hypothesis by comparing the stimulation of the SPL and frontal regions (FEFs or DLPFC) during memory encoding and maintenance. Moreover, ad-hoc paradigms should be used to determine the causal relationship between retro-cueing mechanisms, such as short-term memory capacity freeing, memory trace strengthening, retrieval anticipation and protection from visual interference, and specific regions along the frontoparietal networks.

## 5. Conclusions

In conclusion, by a causal approach with TMS we have shown that online SPL stimulation during encoding reduced the memory capacity in post-cue trials depending on the right SLF-III integrity: the more reduced the right SLF-III integrity, the more decreased the memory capacity in post-cue trials, leaving the performance in retro-cue trials unaffected. This pattern of results further demonstrated that the memory benefit of retrospectively attending to internal representations of previously observed stimuli (the retro-cue benefit) can be altered by TMS over the right SPL at encoding, depending on the individual structural characteristics of long-range white matter connections. No modulations were observed when SPL was stimulated during memory maintenance, thus suggesting that the attention role of SPL is more related to the encoding phase. The present findings provide new information for the better comprehension of the interplay between spatial attention and VSTM, suggesting an important role of SPL during encoding and highlighting the necessity of considering individual differences in the structural characteristics of the white matter connections.

## Data Availability

Data available in a publicly accessible repository that does not issue DOIs. Publicly available datasets were analyzed in this study. This data can be found here: https://osf.io/3s5qd/.

## Supplementary Materials

The following are available online at https://www.mdpi.com/2076-3425/11/2/252/s1. Table S1: Model 1. Type III Analysis of Variance Table with Satterthwaite’s method of the final model found on memory capacity (K) and with the HMOA (hindrance modulated orientational anisotropy) index of both the right and left SLF-III as fixed factors, Table S2: Model 2. Type III Analysis of Variance Table with Satterthwaite’s method of the final model found on confidence ratings and with the HMOA (hindrance modulated orientational anisotropy) index of both the right and left SLF-III as fixed factors, Table S3: Model 3. Type III Analysis of Variance Table with Satterthwaite’s method of the final model found on memory capacity (K) and with the HMOA (hindrance modulated orientational anisotropy) index of both the right and left SLF-I as fixed factors, Table S4: Model 4. Type III Analysis of Variance Table with Satterthwaite’s method of the final model found on confidence ratings and with the HMOA (hindrance modulated orientational anisotropy) index of both the right and left SLF-I as fixed factors.
