# Supplementary material for: Transcranial Magnetic Stimulation of the Right Superior Parietal Lobule Modulates the Retro-Cue Benefit in Visual Short-Term Memory
Source: Brain Sci. 2021 Feb 18;11(2):252. doi: 10.3390/brainsci11020252 (PMC7922694; doi:10.3390/brainsci11020252)
Supplement: Supplementary file 1 [file brainsci-11-00252-s001.pdf]

Table S1. Model 1. Type III Analysis of Variance Table with Satterthwaite's method of the final model found on memory capacity (K) and with the HMOA (hindrance modulated orientational anisotropy) index of both the right and left SLF-III as fixed factors.

Fixed effects	***Mean Sq***	***Num df***	***Den df***	***F value***	***p***
**Cue-Type**	0.06	1	90	.17	.67
**Stimulation Site**	0.23	1	90	.66	.41
**Stimulation Time Window**	0.004	1	90	.01	.91
**SLFR-III**	0.49	1	12	1.41	.25
**SLFL-III**	0.05	1	12	.16	.68
**Cue-Type: Stimulation Site**	1.83	1	90	5.26	*.02
**Cue-Type: Stimulation Time**	0.005	1	90	.01	.89
**Stimulation Site: Stimulation Time**	.17	1	90	.04	.82
**Cue-Type:SLFR-III**	1.59	1	90	4.57	*.03
**Stimulation Site:SLFR-III**	0.22	1	90	.64	.42
**Stimulation Time:SLFR-III**	0.009	1	90	.02	.87
**Cue-Type:SLFL-III**	1.57	1	90	4.51	*0.03
**Cue-Type: Stimulation Site: Stimulation Time**	2.46	1	90	7.06	**.009
**Cue-Type: Stimulation Site: SLFR-III**	1.85	1	90	5.30	*.02
**Cue-Type:Stimulation Time: SLFR-III**	0.019	1	90	.05	.81
**Stimulation Site:Stimulation Time:SLFR-III**	0.003	1	90	.008	.92
**Cue Type:Stimulation Site: Stimulation Time: SLFR-III**	2.48	1	90	7.10	**.009

Table S2. Model 2. Type III Analysis of Variance Table with Satterthwaite's method of the final model found on confidence ratings and with the HMOA (hindrance modulated orientational anisotropy) index of both the right and left SLF-III as fixed factors.

Fixed effects	***Mean Sq***	***Num df***	***Den df***	***F value***	***p***
**Cue-Type**	.04	1	100	1.06	.30
**Stimulation Site**	.25	1	100	6.56	*.01
**SLFR-III**	.05	1	12	1.36	.26
**SLFL-III**	.04	1	12	1.11	.31
**Cue-Type: SLFR-III**	.23	1	100	6.08	*.01
**Cue-Type: SLFL-III**	.52	1	100	13.6	***.0003
**Stimulation Site: SLFL-III**	.28	1	100	7.51	**.007

Table S3. Model 3. Type III Analysis of Variance Table with Satterthwaite's method of the final model found on memory capacity (K) and with the HMOA (hindrance modulated orientational anisotropy) index of both the right and left SLF-I as fixed factors.

Fixed effects	***Mean Sq***	***Num df***	***Den df***	***F value***	***p***
**Cue-Type**	19.9	1	103	52.1	***<.0001
**Stimulation Time Window**	2.85	1	103	7.48	**.007

Table S4. Model 4. Type III Analysis of Variance Table with Satterthwaite's method of the final model found on confidence ratings and with the HMOA (hindrance modulated orientational anisotropy) index of both the right and left SLF-I as fixed factors.

Fixed effects	***Mean Sq***	***Num df***	***Den df***	***F value***	***p***
**Cue-Type**	.11	1	103	2.48	.11
**SLFL-I**	.17	1	13	4.01	.06
**Cue-Type:SLFL-I**	.25	1	103	5.68	*.01
